# 1437. Successful Control of *Candida auris* Outbreak in a Tertiary Hospital Intensive Care Unit in Greece

**DOI:** 10.1093/ofid/ofad500.1274

**Published:** 2023-11-27

**Authors:** Anna Nikopoulou, Paschalia Skalisti, Sophia Tselenkidou, Anastasia Veleni, Sofia Papoti, Theodoros Karampatakis, Helen Katsifa, Christina Iasonidou, Eirini Christaki, Damianos Sotiropoulos

**Affiliations:** G. Papanikolaou General Hospital of Thessaloniki, Thessaloniki, Thessaloniki, Greece; G. Papanikolaou General Hospital of Thessaloniki, Thessaloniki, Thessaloniki, Greece; G. Papanikolaou General Hospital of Thessaloniki, Thessaloniki, Thessaloniki, Greece; G. Papanikolaou General Hospital of Thessaloniki, Thessaloniki, Thessaloniki, Greece; G. Papanikolaou General Hospital of Thessaloniki, Thessaloniki, Thessaloniki, Greece; G. Papanikolaou General Hospital of Thessaloniki, Thessaloniki, Thessaloniki, Greece; G. Papanikolaou General Hospital of Thessaloniki, Thessaloniki, Thessaloniki, Greece; G. Papanikolaou General Hospital of Thessaloniki, Thessaloniki, Thessaloniki, Greece; University General Hospital of Ioannina, Faculty of Medicine, University of Ioannina, Ioannina, Ioannina, Greece; G. Papanikolaou General Hospital of Thessaloniki, Thessaloniki, Thessaloniki, Greece

## Abstract

**Background:**

Candida auris is an emerging pathogen, which can spread efficiently and cause outbreaks in healthcare settings.The purpose of this study is to highlight the importance of strict infection control measures to prevent in-hospital *C. auris* transmission.

**Methods:**

Single-center retrospective observational study in hospitalized patients at the 2nd Department of Intensive Care Medicine (ICU) of G. Papanikolaou General Hospital from 02-11-2022 until 28-02-2023. Patient outcomes and epidemiologic indices were recorded. In total 79 surveillance cultures in 18 patients (male: 75%, median age: 51 years) were performed, while 187 surface samples were collected.

**Results:**

After *C. auris* isolation from the index patient, colonization screening was performed in all ICU patients. *C. auris* was detected in a neighboring patient, 24 hours later, and in another patient from a central venous catheter tip culture 2 months later. An environmental culture from a pulse oximeter, was also positive. Immediately, an epidemiologic investigation was conducted, and an intensive infection control program was implemented in the ICU. Measures included strict adherence to standard and contact precautions and hand hygiene, isolation of positive cases, dedicated staff to care for patients with *C. auris*, disposable or specialized equipment for patient care, and cleaning and disinfection of frequently touched surfaces and equipment. In addition, surveillance cultures were performed twice weekly for each patient and once weekly for the environment. Also, to control the transmission to other departments, patient transfer was limited. At the same time, ICU personnel was educated on the significance and implementation of infection control bundles. The above measures resulted in the control of transmission of *Candida auris* in our hospital.

**Conclusion:**

Strengthening appropriate infection control interventions and increasing compliance to their implementation, combined with continued surveillance and healthcare worker engagement by education, can prevent the transmission of pathogens such as *Candida auris* in healthcare settings.

**Disclosures:**

**All Authors**: No reported disclosures

